# PSMA-Targeting Imaging and Theranostic Agents—Current Status and Future Perspective

**DOI:** 10.3390/ijms23031158

**Published:** 2022-01-21

**Authors:** Sashi Debnath, Ning Zhou, Mark McLaughlin, Samuel Rice, Anil K. Pillai, Guiyang Hao, Xiankai Sun

**Affiliations:** 1 Department of Radiology, University of Texas Southwestern Medical Center, Dallas, TX 75390, USA; Sashi.Debnath@UTSouthwestern.edu (S.D.); Ning.Zhou@UTSouthwestern.edu (N.Z.); Samuel.Rice@UTSouthwestern.edu (S.R.); Anil.Pillai@UTSouthwestern.edu (A.K.P.); Guiyang.Hao@UTSouthwestern.edu (G.H.); 2 Department of Radiation Oncology, University of Texas Southwestern Medical Center, Dallas, TX 75390, USA; Mark.Mclaughlin@UTSouthwestern.edu; 3 Advanced Imaging Research Center, University of Texas Southwestern Medical Center, Dallas, TX 75390, USA

**Keywords:** prostate-specific membrane antigen, positron emission tomography, prostate cancer, prostate-specific antigen, theranostics, inhibitor, binding affinity, radionuclide therapy

## Abstract

In the past two decades, extensive efforts have been made to develop agents targeting prostate-specific membrane antigen (PSMA) for prostate cancer imaging and therapy. To date, represented by two recent approvals of [^68^Ga]Ga-PSMA-11 and [^18^F]F-DCFPyL by the United States Food and Drug Administration (US-FDA) for positron emission tomography (PET) imaging to identify suspected metastases or recurrence in patients with prostate cancer, PSMA-targeting imaging and theranostic agents derived from small molecule PSMA inhibitors have advanced to clinical practice and trials of prostate cancer. The focus of current development of new PSMA-targeting agents has thus shifted to the improvement of in vivo pharmacokinetics and higher specific binding affinity with the aims to further increase the detection sensitivity and specificity and minimize the toxicity to non-target tissues, particularly the kidneys. The main strategies involve systematic chemical modifications of the linkage between the targeting moiety and imaging/therapy payloads. In addition to a summary of the development history of PSMA-targeting agents, this review provides an overview of current advances and future promise of PSMA-targeted imaging and theranostics with focuses on the structural determinants of the chemical modification towards the next generation of PSMA-targeting agents.

## 1. Introduction

### 1.1. Prostate-Specific Membrane Antigen (PSMA) and PSMA-Targeting Agents

Prostate-specific membrane antigen (PSMA) is a type II transmembrane glycoprotein that consists of 750 amino acids and with a molecular weight greater than 100 kD after glycosylation [1]. Despite “prostate” in its name, its expression has been documented not only in the prostate glands but also in non-prostatic tissues including the duodenum, kidney, salivary glands, neuroendocrine system, and proximal renal tubules. It is known that an elevated PSMA expression is associated with poor outcomes including local spread, relapses, and metastasis [1]. The purpose of this review is to examine the history, current state, and future directions of PSMA-targeted imaging and therapy of prostate cancer. The main focus of this review is on PSMA-targeting imaging and theranostic agents derived from small molecule PSMA inhibitors. Given that the current development of the agents has shifted towards the systematic chemical modifications of the linkage between the targeting moiety and theranostic payloads, this review also provides an overview of critical structural determinants of the chemical modifications for the development of next generation PSMA-targeting agents. Of note, many recent review articles are valuable for covering the applications of PSMA-targeted imaging and therapy [2,3,4,5,6].

Prostate cancer is the second most frequent cancer and the sixth leading cause of cancer death among men worldwide in 2021 [7]. The 5-year relative survival rate for localized prostate cancer is 100%, whereas for metastatic prostate cancer it becomes only 30% [7]. In addition, biochemical reoccurrence after initial therapy is frequent. Therefore, timely detection and staging of primary, metastatic, and relapsed prostate cancer is extremely important for prognostication and management of prostate cancer. The standard of care for diagnosis is an ultrasound-guided biopsy, which, however, could miss 21% to 28% of prostate cancer and under-grade 14% to 17% [8]. Currently, guidelines recommend the use of magnetic resonance imaging (MRI) and positron emission tomography/computed tomography (PET/CT) for evaluation of patients with high-risk disease [9]. The widely used multiparametric MRI demonstrates a pooled sensitivity of 89% and specificity of 73% for identifying prostate cancer [10]. Recently, two PSMA-targeting radiotracers have received approval from the United States Food and Drug Administration (US-FDA), [^68^Ga]Ga-PSMA-11 and [^18^F]F-DCFPyl. Particularly favorable in patients with low prostate-specific antigen (PSA) level, both of them provide superior sensitivity and specificity profiles for recurrent or metastatic prostate cancer than the earlier approved PET radiotracers (e.g., [^18^F]fluciclovine and [^11^C]choline).

To date, PSMA has shown several significant advantages over other cell surface markers for prostate cancer imaging and therapy:

- PSMA expression can be elevated to 100- to 1000-fold higher than that in normal tissues [11]. The selective overexpression is also observed in cancerous lymph nodes and bone metastases [12].
- PSMA is expressed by a very high proportion of prostate cancer tumors and at nearly all stages of the disease. In one immunohistochemical (IHC) analysis, PSMA expression was detected in 94% of prostate cancer samples [13]. Furthermore, increased PSMA expression is correlated with an increased tumor grade, pathologic stage, aneuploidy, and/or biochemical recurrence [14].
- The transmembrane conformational structure of PSMA enables it to internalize bound agents by means of endosomal complexes, which is a highly attractive feature for targeted therapies [15].
- PSMA belongs to the enzyme class of carboxypeptidases. The preferred substrate of PSMA is a peptide with a C-terminal glutamate. As such, varieties of small molecule PSMA inhibitors have been developed and radiolabeled with many different radioisotopes.

As an antigen protein and an enzyme, PSMA has served as a target of interest for imaging and therapeutic agent development. In general, PSMA-targeting agents are developed from two categories of targeting moieties: (1) anti-PSMA antibodies or engineered protein fragments and (2) small molecule inhibitors of the enzymatic activity of PSMA.

### 1.2. Monoclonal Antibodies of PSMA for Diagnosis and Therapy

An ^111^In-labeled anti-PSMA monoclonal antibody (mAb), ^111^In-capromab pendetide (ProstaScint), is the first US-FDA-approved radiopharmaceutical agent for the detection of prostate cancer via gamma scintigraphy or single photon-emission computed tomography (SPECT) [16]. It consists of a mAb against the cytoplasmic domain of PSMA (7E11-C5) and an ^111^In chelator incorporated linker, glycyl-tyrosyl-(*N*-ε-diethylenetriamine pentacetic acid)-lysine (GYK-DTPA). However, as the targeted cells are mostly necrotic in nature and 7E11-C5 only recognizes the intracellular epitope of PSMA, SPECT imaging or gamma scintigraphy of prostate cancer with ProstaScint suffers low sensitivity and specificity. For instance, a prospective clinical evaluation published in 1999 reported lymph node detection sensitivity of 62% and specificity of 72% in 152 evaluable patients with prostate cancer who underwent ProstaScint scintigraphy followed by pelvic lymph node dissection [17]. Therefore, efforts had been seen in developing antibodies that bind the extracellular domain of PSMA. Indeed, the antibodies showed improved in vivo binding kinetics. For instance, an anti-PSMA mAb, E6, was reported with binding to the extracellular domains of both mouse and human PSMA and its toxicity was assessed in preclinical studies [18]. Impressively, a humanized anti-PSMA mAb, J591, demonstrated its potential to overcome the drawbacks of ProstaScint [19] when functionalized with chelators for radiolabeling with a variety of radionuclides, including ^90^Y and ^177^Lu for β-therapy [19,20], ^213^Bi and ^225^Ac for α-therapy [21,22], ^111^In and ^99m^Tc for SPECT [23,24], and ^64^Cu and ^89^Zr for PET [25,26]. However, the mAb conjugates cannot be physically cleared from the blood through the glomerular filtration because of their large size, which results in a prolonged blood retention that reduces the tumor-to-background contrast. As such, the major obstacle remains because of the inherent slow clearance of antibodies from non-target tissues.

### 1.3. Small Molecule Inhibitors/Ligands of PSMA

**Figure 1:**
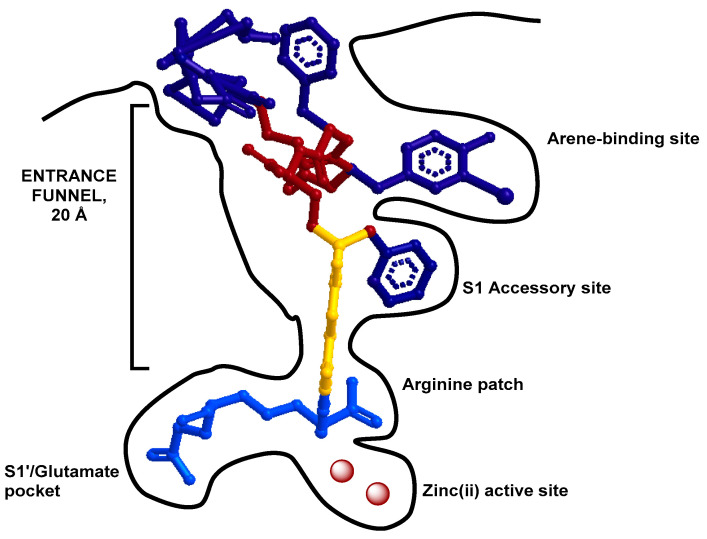
Schematic interactions between a Glu-ureido–based ligand and the PSMA binding cavity [31].

With the advantages of rapid extravasation, quick diffusion in extravascular space, and efficient blood clearance, to date, small molecule inhibitors of PSMA have dominated the development of PSMA-targeting imaging agents, therapies, and/or theranostic agents, particularly for prostate cancer. Also known as human neuropeptidase glutamate carboxypeptidease II (GCP II), PSMA served as a plausible target for the development of small molecule inhibitors for the treatment of neurological disorders [27]. The preferred substrate of PSMA is a peptide with a C-terminal glutamate, which binds to the active binding site. In a potent PSMA inhibitor that binds to the active site, a glutamate motif is essential. To enhance the desired binding affinity and avoid the unwanted dissociation of the bound inhibitor, the structure should not possess an enzyme-cleavable bond. To date, three types of PSMA inhibitors have been reported: (1) phosphorus-based, (2) thiol-based, and (3) urea-based ligands [28]. Phosphorus-based ligands bind via a phosphonate core to binuclear zinc ions positioned in the active PSMA domain (Figure 1). Unfortunately, the inherent high polarity of those ligands limits their ability to penetrate the blood–brain barrier. Therefore, the attention quickly shifted to other types of structures because the purpose of GCP II inhibitor development was for neurological disorders. PSMA ligands with thiol functionality undergo disulfide bond formation, leading to insufficient metabolic stability for clinical application. Among varieties of structures reported to date, the urea-based structure demonstrates the desired high binding affinity and stability. Further optimization also reveals that a linker between the PSMA binding motif and the chelator enables the binding motif to reach the active site through a tunnel (~20 Å) in the PSMA extracellular domain and keep the bulky metal chelate moiety outside (Figure 1) [29]. In addition, it has been reported that negatively charged linkers could reduce off-target retention [30] and the introduction of hydrophobic aromatic structure to the linker offers opportunities to improve the PSMA binding affinity while decreasing kidney uptake.

### 1.4. PSMA-Targeting Radiopharmaceuticals

Despite the success of [^18^F]FDG (2-deoxy-2-[^18^F]fluoro-D-glucose) as a PET probe in other cancer types, its application in prostate cancer is limited by the facts that (1) prostate cancer at early stages is not glucose avid and (2) low imaging quality in prostate glands because [^18^F]FDG is excreted from the bladder nearby. As such, other biological and biochemical mechanisms have been explored for noninvasive imaging of prostate cancer. For instance, [^11^C]choline (US-FDA approval in 2012 for imaging of biochemically recurrent prostate cancer) preferentially accumulates in prostate cancer cells [32] because choline is an essential nutrient and a critical phospholipid precursor for cell surface. Structurally related to l-leucine, [^18^F]fluciclovine (anti-1-amino-3-^18^F-fluorocyclobutane-1-carboxylic acid, US-FDA approval in 2016) is a metabolic PET probe that accounts for amino acid internalization into the cells by L-type amino acid transporter 1 and alanine-serine-cysteine transporter 2 (LAT1/ASCT2) [33]. A comparative study between [^18^F]fluciclovine and [^11^C]choline in patients with biochemically relapsed prostate cancer (N = 89) showed that [^18^F]fluciclovine performed slightly better than [^11^C]choline (sensitivity 37% vs. 32%) [34], whereas both were suboptimal.

**Figure 2:**
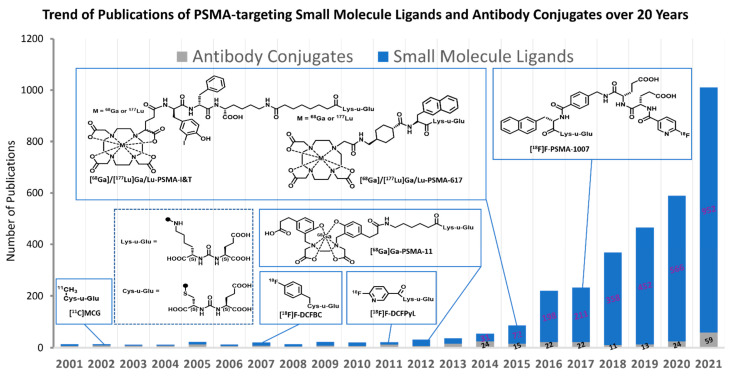
An exponential trend of publications is observed on reporting the chemical modifications and applications of small molecule PSMA-targeting agents since the initial report of [^11^C]MCG in 2002. Data were obtained from SciFinder covering journal publications of structural investigation and pre-clinical/clinical applications of PSMA-targeting small molecules and antibodies in English. Shown in the figure are representative chemical structures and their corresponding first publication year: [^11^C]MCG, [^68^Ga]Ga-PSMA-11, [^68^Ga]Ga-/[^177^Lu]Lu-PSMA-617, [^68^Ga]Ga-/[^177^Lu]Lu-PSMA-I&T, [^18^F]F-DCFBC, [^18^F]F-DCFPyL, and [^18^F]F-PSMA-1007. [^11^C]MCG: [^11^C](S)-2-[3-((R)-1-carboxy-2-methylsulfanyl-ethyl)-ureido]-pentanedioic acid; [^18^F]F-DCFBC: N-[N-[(S)-1,3-dicarboxypropyl] carbamoyl]-4-[^18^F]fluorobenzyl-L-cysteine; [^18^F]F-DCFPyL: 2-(3-(1-carboxy-5-[(6-[^18^F]fluoropyridine-3-carbonyl)-amino]-pentyl)-ureido)-pentanedioic acid.

In the past two decades, tremendous efforts have been undertaken to modify small molecule GCP II inhibitors for PSMA-targeted imaging and therapy development. Figure 2 summarizes the number of publications of PSMA-targeting agents from 2001 to 2021, which covers the journal publications of structural investigation and pre-clinical/clinical applications of PSMA-targeting small molecules and antibodies in English from SciFinder. Particularly stimulated by the success of [^68^Ga]Ga-PSMA-11 (or [^68^Ga]Ga-HBED-CC-Ahx-Lys(OH)-CO-Glu(OH); [^68^Ga]Ga(3S,7S)-22-[3-[[[2-[[[5-(2-carboxyethyl)-2-hydroxyphenyl]-methyl](carboxymethyl)amino]ethyl](carboxymethyl)-amino]-methyl]-4-hydroxyphenyl]-5,13,20-trioxo-4,6,12,19-tetraazadocosane-1,3,7-tricarboxylic acid) and [^18^F]F-DCFPyl (2-(3-(1-carboxy-5-[(6-[^18^F]fluoropyridine-3-carbonyl)-amino]-pentyl)-ureido)-pentanedioic acid), an exponential growth of reported studies in the literature is observed in the recent 5 years on the structure modification and biological evaluation of PSMA-targeting agents.

Prior to the emergence of urea-based PSMA-targeting agents, considerable efforts have been seen on using the phosphorus-based (2-PMPA [35], GPI 5232 [36], GPI 18431 [37]) and thiol-based (2-MPPA [38], E2072 [39]) small molecule PSMA inhibitors. Phosphorus-based PSMA-targeting agents need to overcome the in vivo competition with phosphates. In addition, a reversible internalization profile was observed for phosphorus-based PSMA inhibitors due to the exchange of the P–O bond with phosphoramidate P–N link, which reduces the overall internalization [40]. To overcome the in vivo competition with phosphates, a multivalent presentation on a chelator scaffold of a PSMA targeting moiety, GPI (2[(3-amino-3-carboxypropyl)(hydroxy)(phosphinyl)-methyl]pentane-1,5-dioic acid), was proven effective [41], but remains to be further explored. On the other hand, thiol-based PSMA inhibitors can be easily oxidized in nature, which compromises the in vivo metabolic stability and potentially activates unwanted immune responses [42].

In 2002, the Pomper group firstly reported a ^11^C-labeled urea-based PSMA inhibitor with cysteine and glutamine residues, [^11^C]MCG [43]. [^11^C]MCG displayed the desired specific uptake to PSMA-positive xenografts with tumor-to-muscle ratio up to 11. This initial success caught considerable attention in the field leading to the further development of varieties of PSMA-targeting agents using urea-based PSMA inhibitors. One of the notable breakthroughs was the first-in-human PET imaging with a ^68^Ga-labeled PSMA-targeting agent, [^68^Ga]Ga-PSMA-11 ([^68^Ga]PSMA-HBED-CC or [^68^Ga]DKFZ-PSMA-11 in the literature), reported by the German Cancer Research Center and the University Hospital Heidelberg [44]. This agent is composed of three components: (1) a PSMA-targeting peptidomimetic moiety, Lys-urea-Glu (Lys-u-Glu), (2) an Ahx spacer, and (3) a chelator for ^68^Ga-labeling, HBED-CC. The HBED-CC chelator, which contains an amine-phenol backbone, binds strongly with Ga(III). The two phenolic rings in the HBED-CC chelator, together with the aliphatic spacer Ahx, provide an appropriate lipophilicity without compromising the high binding affinity of Lys-u-Glu to PSMA (K_i_ = 12.1 ± 2.1 nM). With efficient blood clearance, relatively low liver uptake (0.87% ID/g at 1 h post-injection (p.i.)), and high specific uptake in PSMA-expressing tissues and tumor (tumor uptake 7.7% ID/g at 1 h p.i.), [^68^Ga]Ga-PSMA-11 has become the most widely used PSMA-targeting imaging agent. The US-FDA approved its use on 1 December 2020, for PET imaging of biochemically recurrent or metastatic castrate-resistant prostate cancer (mCRPC) [45]. The first prospective multicenter clinical trial of [^68^Ga]Ga-PSMA-11 showed 84% to 92% positive predictive value (PPV) and 75% overall detection rate [46]. The recent phase 3 ProPSMA trial showed improved sensitivity, specificity, and accuracy of [^68^Ga]Ga-PSMA-11 for detecting metastatic disease compared to standard of care CT and bone scan imaging in men with high risk prostate cancer [47]. A further phase 3 trial, PSMA-SRT, looking at the utility of utilizing [^68^Ga]Ga-PSMA-11 to guide early salvage radiation therapy has completed enrollment [48].

Although HBED-CC functions as an ideal chelator for ^68^Ga labeling, it is not capable of forming stable complexes with therapeutic metal radionuclides such as ^177^Lu, ^225^Ac, and ^213^Bi. To enable PSMA-targeted radionuclide therapies, PSMA-617 was designed with a DOTA chelator (DOTA: 2,2′,2″,2‴-(1,4,7,10-tetraazacyclododecane-1,4,7,10-tetrayl)tetraacetic acid) and a naphthylic spacer but with the same PSMA-targeting moiety, Lys-u-Glu [29]. In addition, it forms stable complexes with ^68^Ga and ^64^Cu. Tumor uptake levels of [^68^Ga]Ga-PSMA-617 and [^177^Lu]Lu-PSMA-617 were 8.5% ID/g and 11.2% ID/g, respectively [49]. In general, [^177^Lu]Lu-PSMA-617 showed optimal key parameters for therapeutic radiopharmaceutical development, such as stronger PSMA binding affinity, more internalization, higher tumor-to-background contrast at late time points, and faster kidney clearance than [^68^Ga]Ga-PSMA-11. One recent review of 17 clinical studies with [^177^Lu]Lu-PSMA-617 therapy for the treatment of progressive metastatic prostate cancer reported that the majority of patients responded to the PSMA-targeted radionuclide therapy and the survival was found to be upwards of one year [50]. Other clinical trials with [^177^Lu]Lu-PSMA-617 are noteworthy:

The phase 2 trial comparing [^177^Lu]Lu-PSMA-617 versus cabazitaxel in patients with metastatic castration-resistant prostate cancer (theraP), phase 2 trial, found that treatments with [^177^Lu]Lu-PSMA-617 had fewer grade 3/4 adverse events (33% vs. 53%) and a higher PSA response rate (66% vs. 37%) [51].

The Phase 3 VISION trial, which compared the standard of care with the standard of care plus [^177^Lu]Lu-PSMA-617 in patients with mCRPC- and [^68^Ga]Ga-PSMA-11-positive PET scans who progressed after both taxane and novel androgen axis therapy. With a median follow up of 20.9 months, the [^177^Lu]Lu-PSMA-617 arm demonstrated superior progression free survival (8.7 months vs. 3.4 months) and overall survival (15.3 months vs. 11.3 months) [52].

The UpFrontPSMA trial is an ongoing phase 2 trial which examines the inclusion of [^177^Lu]Lu-PSMA-617 in the initial treatment of de novo hormone naïve prostate cancer. Patients are randomized between [^177^Lu]Lu-PSMA-617 plus standard of care androgen therapy and docetaxel vs. androgen therapy and docetaxel alone with the primary endpoint of undetectable PSA at one year [53].

PSMA-I&T (I&T stands for Imaging and Therapy) represented another series of ligands including DOTA, a Lys-u-Glu targeting motif, and a D-amino acid-substituted peptidyl spacer. In patients with mCRPC who had failed chemotherapy and a novel androgen receptor targeted therapy, [^177^Lu]Lu-PSMA-I&T showed the expected treatment efficacy [54], with 68% showing stable or improved disease, whereas 30% showed a decline in PSA levels.

Based on the initial success of [^11^C]MCG, the Pomper lab replaced ^11^C with ^18^F for more desirable clinical accessibility of the PSMA-targeting agents, which resulted in the first generation agent, [^18^F]F-DCFBC in 2008 [55], and then the second generation agent, [^18^F]F-DCFPyL in 2011 [56]. [^18^F]F-DCFPyL contains an [^18^F]fluoropyridyl-substituted Lys-u-Glu motif, different from the [^18^F]fluorobenzyl-substituted Cys-u-Glu motif in [^18^F]F-DCFBC. These structural changes succeeded in (1) improving tumor uptake (39.4 %ID/g vs. 4.7% ID/g at 2 h p.i.), (2) reducing kidney accumulation (7.4% ID/g vs. 13% ID/g at 2 h p.i.), and (3) facilitating clearance from non-target tissues (blood 0.03% ID/g vs. 0.4 %ID/g at 2 h p.i.). The CONDOR trial, a prospective multicenter study designed to evaluate the diagnostic performance of [^18^F]F-DCFPyL, demonstrated that the radiotracer correctly localized disease in 85% of 208 men with biochemically recurrent prostate cancer [57]. Of the evaluable patients with positive [^18^F]F-DCFPyL PET/CT findings, 73% underwent a change in intended management after the scan, including salvage local therapy to systemic therapy (28%), noncurative systemic therapy to salvage local therapy (21%), and observation to initiating therapy (24%). This clearly indicates the clinical impact of the PSMA-targeted PET scans on cancer patient care. A retrospective matched pair analysis of patients with biochemically recurrent or relapsed prostate cancer comparing patients scanned with [^18^F]F-DCFPyL or [^68^Ga]Ga-PSMA-11 indicated that [^18^F]F-DCFPyL had higher sensitivity (88% vs. 66%) for PSA values between 0.5 and 3.5 μg/L and similar sensitivity otherwise [58].

The ^18^F-labeled DKFZ-PSMA-617, also known as [^18^F]F-PSMA-1007, had a modified spacer from the PSMA-617 structure [59]. The introduction of two glutamic acid residues and a 6-[^18^F]fluoronicotinic acid increased hydrophilicity. [^18^F]F-PSMA-1007 showed exceptionally high tumor cell internalization (67% ± 13%) in vitro and high tumor uptake (8.0% ID/g at 1 h p.i.) in vivo. A pilot crossover clinical trial of [^18^F]F-PSMA-1007 and [^18^F]F-DCFPyL showed comparable detection sensitivity and specificity of the two agents for local tumor and pelvic lymph node metastasis in high risk, treatment-naïve patients [60]. Another prospective head-to-head comparison of [^18^F]fluorocholine and [^18^F]F-PSMA-1007 PET/CT in 40 patients with biochemically relapsed prostate cancer (PSA < 2.0 ng/mL) demonstrated a clear advantage of [^18^F]F-PSMA-1007 over [^18^F]fluorocholine for the detection of recurrent lesions (60% vs. 5%), as determined by trained nuclear medicine physicians [61].

## 2. Current Status of PSMA-Targeting Agents

To date, varieties of urea-based PSMA-targeting agents have been reported for imaging, radiotherapy, or theranostic applications when labeled with imaging and therapeutic radionuclides. Some of them have moved to clinical trials. For theranostic applications, the current practice is through a sequential administration of diagnostic and therapeutic radiopharmaceuticals, where the diagnostic one provides guidance to oncologists before the treatment. In such a sequential practice, the chemical differences between the diagnostic and therapeutic pair, which likely give rise to different in vivo kinetic profiles, may compromise the purpose of theranostic strategies for precision treatment [62]. On the other hand, we have started seeing efforts to tackle the problem by developing a concurrent theranostic strategy, where an identical theranostic pair of agents employed for simultaneous diagnosis and therapy without altering the in vivo distribution and kinetics [63]. For the development of more efficacious PSMA-targeting agents (measured by higher specific PSMA binding and more favorable in vivo kinetics), many structural determinants need to be considered in the chemical modification or novel design, such as charge, length, chemical components, hydrophilicity vs. lipophilicity, etc. In the meantime, the accessibility of the whole conjugate should not be obstructed to the active site through the funnel (~20Å) in PSMA (Figure 1) [64].

### 2.1. Radiometal-Based PSMA-Targeting Agents beyond [^68^Ga]Ga-PSMA-11 and [^177^Lu]Lu-PSMA-617

**Figure 3:**
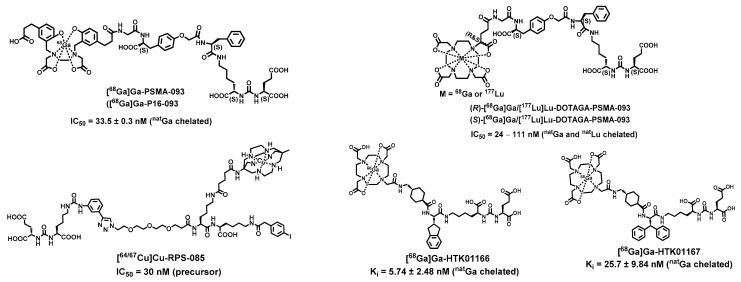
Representative radiometal-based PSMA targeting agents [67,68,69,70].

Despite the success of the theranostic pair of [^68^Ga]Ga-PSMA-11 and [^177^Lu]Lu-PSMA-617 in clinical trials and practice, further chemical modifications are required to alleviate side effects while improving the treatment efficacy because (1) the performance of [^68^Ga]Ga-PSMA-11 in prostate cancer local recurrence is suboptimal due to its rapid in vivo kinetics and high renal uptake, and (2) [^177^Lu]Lu-PSMA-617 is more tuned for radionuclide therapy with slower in vivo kinetics to enhance tumor uptake and lower renal accumulation to reduce the kidney toxicity [65,66]. Given the structures of the agents, the current efforts are focused on chemical modifications of the linker between the chelator and the PSMA-target moiety without compromising the PSMA-specific binding affinity. Reported structural modifications (Figure 3) are summarized below:

#### 2.1.1. Incorporation of Amino Acids into the Linkage

[^68^Ga]Ga-PSMA-093 (Figure 3) was developed by the incorporation of a specific linker *O*-(carboxymethyl)-l-tyrosine between the Lys-u-Glu and HBED-CC. The HBED-CC chelator with the amine-phenol backbone forms a thermodynamically stable complex with ^68^Ga(III) (*K_a_*~10^39^). The combination of HBED-CC containing two phenolic rings and the linker *O*-(carboxymethyl)-l-tyrosine provides [^68^Ga]Ga-PSMA-093 with an optimal lipophilicity for higher PSMA binding affinity and superior in vivo pharmacokinetics [67]. Indeed, [^68^Ga]Ga-PSMA-093 showed a prostate cancer detection capability similar to [^68^Ga]Ga-PSMA-11 in PET but with less urinary bladder excretion.

#### 2.1.2. Stereochemistry of the Linkage

The better suitability of DOTA derivatives for ^177^Lu(III) complexation offers the opportunities of incorporating amino acids into the linkage for the development of PSMA-targeting imaging and theranostic agents. For instance, an extra glutamic arm imparts more stable ^68^Ga or ^177^Lu complex moiety to DOTAGA derivatives (PSMA I&T) along with higher specific binding and superior in vivo pharmacokinetics [54]. However, because of the amino acid incorporation, one might expect that the optical purity of the linkage would play a role in the design and development of PSMA-targeting agents. Actually, the use of (D)-amino acid is a common practice in the field of drug design for higher metabolic stability and more effective clearance from off-targets [71]. To evaluate the potential effects of the stereochemistry of the linkage on PSMA I&T, optically pure enantiomers, (*R*)- and (*S*)-[^68^Ga]Ga/[^177^Lu]Lu-DOTAGA-PSMA-093 (Figure 3), were developed [68]. Although both conjugates showed similar PSMA-binding and tumor accumulation, which is consistent with a literature report [72], radiolabeling with ^177^Lu occurred quicker (~1.5 time) with (*S*)-DOTAGA-PSMA-093 than with (*R*)-DOTAGA-PSMA-093. Given the half-life of ^177^Lu, this difference in labeling kinetics, however, is not expected to play a significant role in future development of PSMA-targeting agents.

#### 2.1.3. Lipophilicity vs. Hydrophilicity of the Linkage

Given that there is an S1-accessory hydrophobic pocket in PSMA, aryl functionalities can be leveraged to enhance the PSMA-binding affinity for structure-aided PSMA targeting agent design (Figure 1). In addition, the incorporation of aryl functionalities into the linkage modulates the hydrophobicity of the agents thus influencing their in vivo kinetics. To evaluate the structure–activity relationship, hydrophobic linker modifications were performed on the same basic chemical construct, which demonstrates that (1) multiple aromatic rings in the linker fragment improved the hydrophobic interaction with S1-accessory pocket suitable for PSMA-specific cell surface binding and cell internalization, (2) a rigid aromatic modification (2-naphthyl-l-alanine) in the linker benefited the PSMA-specific cell internalization, and (3) incorporation of 2-naphthyl-l-Ala-AMCH showed an optimal tumor-to-background ratio [73].

Another report further strengthened the design concept of PSMA-targeting agents by optimizing the structure’s interactions with the S1-accessory hydrophobic pocket of PSMA (Figure 1). Replacement of 2-naphthyl-l-alanine with 2-indanylglycine (Igl) or 3,3-diphenylalanine (Dip) demonstrated that the hydrophobic interaction with the S1 hydrophobic pocket of PSMA can be leveraged to improve the desired binding affinity (Igl substitution: K_i_~5.74 nM vs. Dip substitution: K_i_~25.7 nM) [70].

#### 2.1.4. Incorporation of a Serum Albumin Binding Moiety into the Linkage

Small molecules typically showed rapid in vivo distribution and clearance. Therefore, it is necessary to balance the physical half-life and the biological half-life of the small molecule based PSMA-targeting agents. Incorporation of an albumin binding motif has proven to be an effective strategy to achieve the desired in vivo kinetics of theranostic molecules or conjugates: rapid blood clearance and quick tissue distribution together with progressive uptake and sustained retention in tumors. For instance, incorporation of N^ε^-(2-(4-iodophenyl)acetyl)lysine, a motif that binds serum albumin weakly (*K_d_* = 9.9 ± 1.7 μM), into the structure of [^64^/^67^Cu]Cu-RPS-085 (Figure 3) enhanced the tumor accumulation while facilitating the renal excretion of the PSMA-targeting agents [69].

### 2.2. Fluorine-18 Labeled Diagnostic PSMA-Targeting Agents

**Figure 4:**
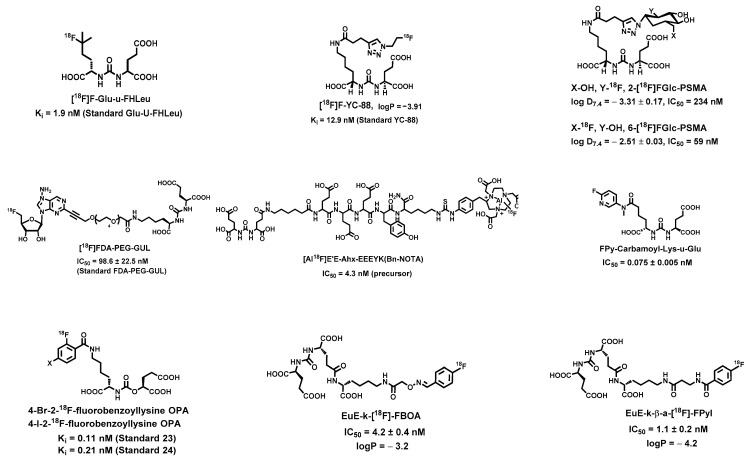
Representative ^18^F-labeled diagnostic PSMA-targeting ligands [30,31,77,78,79,80,81,82].

Although head-to-head comparisons are lacking between the clinical utility of [^68^Ga]Ga-PSMA-11 and [^18^F]F-DCFPyL, it is believed that [^18^F]F-DCFPyL possesses advantages over [^68^Ga]Ga-PSMA-11 simply because of the physical decay properties of ^18^F (t_1/2_ = 109.7 min, 97% β^+^, E_β+-max_ = 0.63 MeV) vs. ^68^Ga (t_1/2_ = 67.7 min, 89% β^+^, E_β+-max_ = 1.92 MeV). In addition, the high yield of ^18^F production enables a network distribution of [^18^F]F-DCFPyL to local hospitals similar to the commercial distribution of [^18^F]FDG. Although the solid-target production of [^68^Ga]Ga-PSMA-11 has been recently reported [74], the current dose volume availability of [^68^Ga]Ga-PSMA-11 or other [^68^Ga]Ga radiopharmaceuticals is limited to onsite clinical practice because of the capability of ^68^Ge/^68^Ga generator or the low yield liquid-target cyclotron production. Therefore, in addition to [^18^F]F-DCFBC, [^18^F]F-PSMA-1007, and [^18^F]F-DCFPyL, many other ^18^F-labeled PSMA-targeting agents have been developed (Figure 4) with the aims to further improve the tumor detection accuracy [75,76]. Furthermore, the strongest bond in organic chemistry, [^18^F]F-C, can also be leveraged to benefit imaging quality by enhancing the in vivo stability of the radiolabel. Main chemical modifications are summarized below:

#### 2.2.1. Minimally Modified Lys-u-Glu Ligands for PSMA-Targeting Agent Design

To take advantage of the direct interaction of ligands containing urea-Glu with S1′ pocket (Figure 1) for high specific PSMA binding and PSMA-mediated internalization for drug delivery, several attempts have been reported with minimally modified structures. Notable examples include [^18^F]F-Glu-u-FHLeu [77] and [^18^F]F-YC-88 (Figure 4) [78], both of which contain the basic unit of urea-Glu that fits in the S1’ pocket whereas the other end was minimally altered for labeling with ^18^F. Impressively, [^19^F]F-Glu-u-FHLeu showed a PSMA binding affinity (K_i_ = 1.9 nM) similar to [^18^F]F-DCFBzL (K_i_ = 0.19 nM) and [^18^F]F-DCFPyL (K_i_ = 1.1 nM), which warrants further preclinical evaluation before it can be considered for translational studies. On the other hand, the radiosynthesis of [^18^F]F-YC-88 could be readily carried out in a rapid one-pot click reaction between [^18^F]fluoroethyl azide and an alkyne-containing precursor. Since no ester hydrolysis or intermediate purification was required for the radiosynthesis of [^18^F]F-YC-88, it affords synthetic advantages over [^18^F]F-DCFBzL and [^18^F]F-DCFPyL. In addition, small animal PET imaging of [^18^F]F-YC-88 in mice showed an uptake ratio of 170:1 for PSMA^+^ PC3-PIP to PSMA^−^ PC3-flu tumor xenografts. Although the PSMA-specific binding affinity [^18^F]F-YC-88 is one order of magnitude lower than those of its parent compounds, [^18^F]F-DCFBzL and [^18^F]F-DCFPyL, a head-to-head comparison of [^18^F]F-DCFPyL with [^18^F]F-YC-88 demonstrated reduced accumulation of [^18^F]F-YC-88 in major non-target tissues including the liver, kidneys, and spleen, which resulted in a high tumor/kidney ratio (4:1), likely due to the higher hydrophilicity (logP = −3.91. For comparison, the logP of [^18^F]F-DCFPyL is −3.27) [78].

#### 2.2.2. Glycosylation of PSMA-Targeting Agents

Glycosylation, a common method used to modulate the targeting drug delivery [83,84] has been used to optimize the lipophilicity, in vivo kinetics, and tumor-targeting properties of ^18^F-labeled PSMA-targeting agents. A report with two ^18^F-fluoroglycosylated urea-based PSMA agents, 2-[^18^F]FGlc-PSMA and 6-[^18^F]FGlc-PSMA (Figure 4), demonstrated that incorporation of an ^18^F-labeled glycosyl moiety increases the hydrophilicity thus improving the tumor-to-kidney ratio [79]. It is noteworthy that the overall performance of the agents also depends on the positioning of ^18^F in the glycosyl moiety. Although both 2-[^18^F]FGlc-PSMA and 6-[^18^F]FGlc-PSMA displayed 2- to 3-fold higher tumor uptake than [^68^Ga]Ga-PSMA-11 in a direct comparison study, it was 6-[^18^F]FGlc-PSMA that showed a 10-fold lower kidney accumulation with rapid clearance through the urinary tract. This observation may find application of proper glycosylation for the therapeutic agent design, where low kidney accumulation and rapid renal clearance are essential to minimize the unwanted toxicity.

#### 2.2.3. Incorporation of 5′-Fluorodeoxy-Adenosine into PSMA-Targeting Agents

A more effective and practical radiolabeling with ^18^F is always desirable for the development of ^18^F-labeled PSMA-targeting agents. Introduction of a 5′-fluorodeoxy-adenosine unit to the structure of a PSMA-targeting agent is to take advantage of the fluorinase enzyme for a clean enzymatic radiosynthesis of the ^18^F-labeled agent. This strategy is straightforward as seen in a report by coupling a 5′-chlorodeoxy-adenosine unit with Lys-u-Glu through a polyethylene glycol (PEG) linker [80]. In the presence of fluorinase, the synthesized chlorinated precursor was readily labeled with ^18^F at room temperature in a neutral pH aqueous solution. The obtained PSMA-targeting agent, [^18^F]FDA-PEG-GUL (Figure 4), showed a reasonably high binding affinity to PSMA. Further preclinical evaluation of this agent remains to be seen.

#### 2.2.4. Effect of Highly Negatively Charged Linkers

It is a common practice to use highly negatively charged linkers to suppress the unwanted off-target accumulation of drug conjugates [85]. In addition to the optimization of in vivo kinetics, the incorporation of a long highly negatively charged linker into the design of PSMA-targeting agents can be leveraged for radiolabeling with metal radionuclides or ^18^F via a NOTA chelator addition. For instance, the conjugation of a variety of highly negatively charged linkers including a moiety of E′E-Ahx-EEEYK(Bn-NOTA) to Lys-u-Glu yielded PSMA-targeting constructs (Figure 4), which can be readily labeled with ^18^F after loaded with Al(III) [30]. The preclinical evaluation of the constructs demonstrated that highly negatively charged linkers can be used to minimize non-specific binding and decrease the overall background without compromising the specific binding affinity to PSMA.

#### 2.2.5. Effects of Linker Lengths and Aromatic Substitution on PSMA-Targeting Agents

The PSMA binding cavity can be divided into the nonprime (S1) and prime (S1′) regions, whereas the latter is associated with two Zn^2+^ ions (Figure 1). The flexible nonprime region contains an S1 accessory hydrophobic pocket, which could be exploited for the development of new PSMA inhibitors with high affinity. A series of PSMA inhibitors have been developed based on the 2-aminoadipic acid building block with different linker lengths and N-substituent to enhance the regional hydrophobic interaction with the S1 accessory pocket while maintaining or increasing the overall hydrophilicity for optimal in vivo kinetics [31]. For instance, a highly potent PSMA inhibitor (IC_50_ = 0.075 ± 0.005 nM), (((S)-1-carboxy-5-((6-fluoropyridin-3-yl)(methyl)amino)-5-oxopentyl)-carbamoyl)-l-glutamic acid (FPy-Carbamoyl-Lys-u-Glu), was obtained by the *cis*-amide conformational arrangement of fluorinated pyridine ring and *N*-methyl group, which allows a deep proximity of the inhibitor into the S1 accessory pocket (Figure 1 and Figure 4). Along the same lines, ^18^F-labeled carbamate based PSMA inhibitors, 4-bromo-2-[^18^F]fluorobenzoyllysineoxypentanedioic acid carbamate (4-Br-2-^18^F-fluorobenzoyllysine OPA), and 4-iodo-2-[^18^F]fluorobenzoyllysineoxy-pentanedioic acid carbamate (4-I-2-^18^F-fluorobenzoyllysine OPA), were found with increased PSMA binding affinities due to the optimal interaction of 4-bromo/iodo-2-fluorobenzoyl groups with the arginine patch and S1 accessory pocket [81]. In another report, the evaluation of ^18^F-labeled Glu-u-Glu-(EuE)-based PSMA-targeting agents, EuE-k-[^18^F]-FBOA and EuE-k-*β*-a-[^18^F]-FPyl, showed that FPyl substitution significantly increased the overall hydrophilicity and the PSMA-binding affinity (IC_50_: 1.1 ± 0.2 nM) compared with its FBOA counterpart [82].

### 2.3. PSMA-Targeting Radiotheranostic Agents

In addition to serving as a diagnostic biomarker, PSMA has become a target of choice for radionuclide therapy (RNT). With structures similar to [^177^Lu]Lu-PSMA-617, PSMA-targeted RNT agents are an exhibition of the medical uses of therapeutic radionuclides, namely β- or α-emitters, in tandem with their imaging counterparts for precision prostate cancer treatment via the concept or strategy of theranostics. To maximize the therapeutic efficacy of RNT, the decay half-life, particle emission range, and relative biological effectiveness of the radionuclide of choice should be matched appropriately with tumor’s median size, radiosensitivity, and heterogeneity [86]. In general, β-emitting radionuclides have a relatively long emission range (0.05–12 mm) and a lower linear energy transfer (LET~0.2 keV/µm), which make them more effective in treating medium-to-large tumors [86]. In comparison, with much higher LET (~80 keV/µm) and shorter emission range (40–100 µm), α-emitters are radionuclides of choice for microscopic tumor cell clusters [86].

One common example of theranostics is the radiotheranostic pair of [^68^Ga]Ga-PSMA-11 and [^177^Lu]Lu-PSMA-617 in current clinical trials and practice. The PSMA-targeted PET scans enabled by [^68^Ga]Ga-PSMA-11 are used to stratify patients, to whom [^177^Lu]Lu-PSMA-617 would be administrated or skipped based on the imaging results from individual PET scan. In addition, PSMA-targeted PET scans can be performed in the course of [^177^Lu]Lu-PSMA-617 treatment to monitor the status of PSMA expression for further personalized therapy. Despite the success of the radiotheranostic pair in clinical trials, they differ in radionuclides and compound structures, which inevitably leads to different in vivo kinetics of the agents that result in discrepancies between the imaging-guided RNT expectation and the actual therapy outcome. Development of other small molecule PSMA targeting agents such as MIP-1095 [87], PSMA I&T [88], and PSMA-617 [29] introduced the same chelating moiety, which permits the coordination chemistry with either a diagnostic radionuclide (e.g., ^68^Ga) for imaging or a therapeutic radionuclide (e.g., ^177^Lu, ^90^Y, ^225^Ac) for RNT. These agents possess more compatible in vivo pharmacokinetics for theranostic applications. Another radiotheranostic approach is to take advantage of the available radionuclide pairs for both imaging and therapy, for instance, ^86^Y/^90^Y, ^64^Cu/^67^Cu, ^124^I/^131^I, and ^123^I/^131^I. As these radioisotope pairs are chemically identical, the same molecular constructs can be radiolabeled with one or the other to enable the chemically identical pairs of theranostic agents [89,90]. However, the physical decay half-lives of the radioisotope pairs are different. The actual use of these chemically identical pairs of theranostic agents is often hampered by the shorter half-life of the imaging radioisotope (e.g., ^86^Y, t_1/2_ = 14.7 h), which is incapable of monitoring the RNT course determined by its therapeutic partner (e.g., ^90^Y, t_1/2_ = 2.7 d).

#### 2.3.1. PSMA-Targeted β-RNT

As summarized in Section 1.4, [^177^Lu]Lu-PSMA-617 is the most widely used PSMA-targeting β-RNT agent in clinical trials with promising results. Recently, a multicenter study performed in 145 mCRPC patients for [^177^Lu]Lu-PSMA-617 TRT revealed that PSA level decreased in 45% of patients after 4 cycles and 40% after one cycle [91]. In phase 2 clinical trials (RESIST-PC, NCT03042312) of [^177^Lu]Lu-PSMA-617, 26% of patients exhibited a ≥50% PSA decline after 2 cycles, 37% showed PSA decline at any time, and 21% experienced ≥ 90% PSA decline. Furthermore, a systemic clinical evaluation of [^177^Lu]Lu-PSMA-I&T in patients with mCRPC showed a 30% reduction in serum PSA concentration in 47% of patients [54]. Overall data showed partial or complete responses in 5%, stable disease in 63%, and progressive disease in 32% of patients. Although the tracer accumulation was increased in the kidney and parotid glands, up to 4 cycles of RNT [^177^Lu]Lu-PSMA-I&T showed minimal toxicity.

It is noteworthy that incorporation of an Evans blue (EB) moiety to PSMA-617 (EB-PSMA-617) [92] was proven effective to prolong the blood circulation half-life of ^177^Lu]Lu-EB-PSMA-617, thus increasing the desired tumor uptake and internalization. To date, a clinical trial with [^177^Lu]Lu-EB-PSMA-617 has started in patients with mCRPC [93]. The accumulated radioactivity of [^177^Lu]Lu-EB-PSMA-617 was about 3.02-fold higher than that of [^177^Lu]Lu-PSMA-617. The follow-up PET imaging with [^68^Ga]Ga-PSMA-617 showed a significantly greater tumor uptake reduction within a month in the treatment group of [^177^Lu]Lu-EB-PSMA-617 than in that of [^177^Lu]Lu-PSMA-617 (SUV change: −32.43  ±  0.14% vs. 0.21  ±  0.37%; *p*  =  0.002).

As an added advantage, ^177^Lu also emits gamma (γ) rays, which enables SPECT imaging for radiation dose estimation of therapeutic [^177^Lu]Lu RNT agents [94]. It was reported that the ideal imaging window was within 2–3 days after administration of [^177^Lu]Lu-PSMA-617 for accurate radiation dose estimation across tissue types [94]. Given that the radiation dosimetry is a robust metric to inform the ongoing patient management for personalized treatment optimization, this is of clinical significance in light of the wide accessibility of SPECT scanners in clinic.

Other recent reported PSMA-targeted β-RNT agents in clinical trials include [^131^I]I-MIP-1095 (NCT03939689), [^177^Lu]Lu-PSMA-R_2_ (NCT03490838), whereas many others are still in preclinical development. Notable ones include [^47^Sc]Sc-DOTA-folate and [^90^Y]Y-PSMA-617.

#### 2.3.2. PSMA-Targeted α-RNT

To date, PSMA-targeted α-RNT has shown the anticipated treatment benefits in clinical cases, where the role of [^177^Lu]Lu-PSMA-617 was limited. For instance, [^225^Ac]Ac-PSMA-617 (^225^Ac: t_1/2_ = 9.92 days, 100% α, E_α total_ = 27.9 MeV) was reported with significant antitumor effects but without significant hematologic toxicity in patients with mCRPC [95]. The advantages of [^225^Ac]Ac-PSMA-617 over [^177^Lu]Lu-PSMA-617 in treating these patients are conferred by the substantially higher LET of α-particles over a very short distance thus resulting in a much greater relative biological effectiveness and lower off-target toxicity as compared with β-particles. In addition, unlike β-particles, the lethality of α-particles is independent of the active cell cycle or oxygenation. It was reported that the DNA damage caused by α-particles is much harder to repair than that resulting from β-particles [96].

It is noteworthy that a few other α-emitters (e.g., ^223^Ra: t_1/2_ = 11.43 days, 100 % α, E_α total_ = 26.8 MeV; ^211^At: t_1/2_ = 7.21 h, 100 % α, E_α total_ = 6.9 MeV; and ^213^Bi: t_1/2_ = 45.59 min, 2.09 % α, E_α total_ = 8.5 MeV) have been seen in the development of PSMA-targeted α-RNT, such as [^211^At]At-VK-02-90-Lu [97], [^213^Bi]Bi-PSMA-I&T [98], [^213^Bi]Bi-PSMA-617 [99]. Their preclinical outcomes have been summarized elsewhere [100].

### 2.4. PSMA-Targeted Chemotheranostics

Although toxic to normal cells, conventional chemotherapy remains the standard of care treatment for many cancer types. To mitigate its systemic toxicity, targeted therapy is a straightforward approach of choice. Among the cancer biomarker that have been used for targeted anti-tumor drug delivery, PSMA has served as a productive target. For instance, many types of PSMA-targeting molecules, such as aptamers [101], mAbs, and small molecule inhibitors, have been used in the design and development of PSMA-targeted therapy. To date, several PSMA-targeting antibody drug conjugates (ADCs), such as the J591 conjugated with monomethyl auristatin E (MMAE) [102], have been reported with promising results in both preclinical and clinical studies. Notably, an anti-PSMA mAb conjugated with MMAE through a valine-citruline linker, which was firstly reported in 2006 [103], has entered phase I and II clinical trials [104,105,106,107]. However, as described in Section 1.2, the application of ADC-based PSMA-targeted therapies is hampered by their (i) suboptimal in vivo distribution [108,109], (ii) immunogenicity responses [110], (iii) slow clearance from non-target organs [111,112], and (iv) batch-to-batch manufacturing variation.

With known chemical structures and small sizes, small-molecule drug conjugates (SMDCs) hold promises to overcome these limitations. SMDCs exhibit faster in vivo distribution and kinetics, more efficient penetration into solid tumors, and less adverse immunogenic responses. Moreover, they are single synthetic entities with absolute production reproducibility and low manufacturing cost. For instance, the Pomper lab reported a non-radioactive prodrug, SBPD-1, consisting of a small-molecule PSMA-targeting ligand, a cleavable linker, and MMAE [113]. The prodrug showed high binding affinity to PSMA (K_i_  =  8.84 nM, IC_50_  =  3.90 nM) and significantly reduced toxicity as compared with free MMAE.

Based on the SMDC strategy for PSMA-targeted drug delivery and the concept of theranostics, recently, a unique small molecule theranostic platform was reported to exploit the potential of concurrent theranostic strategy with an identical theranostic pair, [^68/nat^Ga]Ga-NO3A-DM1-Lys-Urea-Glu, for simultaneous therapy and imaging [63]. The small molecule theranostic platform consists of three functional components: (1) a PSMA-targeting ligand (Lys-u-Glu), (2) a highly cytotoxic drug, maytansine (DM1), and (3) a Ga(III)-chelating NO3A moiety for radiolabeling with ^68^Ga or loading with ^nat^Ga. Preclinical PET imaging studies demonstrated the anticipated high and specific uptake of [^68^Ga]Ga-NO3A-DM1-Lys-Urea-Glu in PSMA^+^ PC3-PIP tumors. Although further development of this theranostic design concept is ongoing, it is notable that [^68/nat^Ga]Ga-NO3A-DM1-Lys-Urea-Glu might undergo in vivo demetallation. The release of ^nat^Ga^3+^ ion would form insoluble hydroxide complexes [114,115], which could induce renal toxicity. In addition, the highly hydrophilic nature of ^nat^Ga-NO3A moiety increases the overall size and hydrophilicity of the theranostic pair, which might impede the intended delivery, particularly the intracellular delivery, to cancer cells. Replacement of the Ga(III)-chelating NO3A moiety with a fluorination functionality could offer a potential solution to these limitations.

The design of PSMA-targeted chemotheranostics can also be realized on nano-platforms. Indeed, such PSMA-targeted nanotheranostic agents have been reported. For instance, a hyperbranched polymeric nanocarrier (HBPE) was used to facilitate the selective delivery of a therapeutic peptide (CT20p) that targets and inhibits the chaperonin-containing T-complex protein I after conjugated with PSMA-targeting folic acid and a fluorescent dye for imaging [116]. Notably, lipid nanoparticles can serve as a versatile nano-platform to construct PSMA-targeted nanotheranostic agents by incorporating the desired imaging and therapy functionalities together or separately [117]. These nanotheranostic agents in general fulfil the design concept of chemo-theranostics. However, they face similar hurdles that ADCs encounter when being considered for translational applications.

## 3. Conclusions

In the past decade, we have witnessed an unprecedented development of PSMA-targeted imaging and therapy. The rapid translation of the newly emerged PSMA-targeting imaging and theranostic agents into first-in-human trials is largely driven by the success of [^68^Ga]Ga-PSMA-11 and [^18^F]F-DCFPyL in clinical trials and practice together with the proven clinical value of [^177^Lu]Lu-PSMA-617 in the subsequent treatment of patients with mCRPC. To date, PSMA-targeted imaging and therapy have significantly changed the landscape of prostate cancer patient management, further propelling the development of theranostic radiopharmaceuticals, not only for prostate cancer but also for other cancer types or diseases.

Although numerous comparative studies of existing PSMA-targeting agents vs. other modalities or combinations are ongoing in clinical trials, tremendous efforts are still active toward novel structural designs for higher specific binding affinity and optimal in vivo kinetics for different purposes. Among the functionalities essential to PSMA-targeting properties, the Glu-ureido core stands out as the structural requirement without any other alternatives. Therefore, while keeping the Glu-ureido core unaltered, the design of new PSMA-targeting agents relies on a variety of structural determinants in the linkage for better non-covalent bonding interactions within the entrance funnel of PSMA and balanced hydrophilic/lipophilic modifications for optimal in vivo behavior required for imaging, therapeutic, or theranostic agents. More recently, we have seen a considerably upward trend of PSMA-targeted RNT development, which includes the use of both β- and α-emitters. Because of the substantially different LET of α-particles vs. β-particles, the developments of PSMA-targeted α- and β-RNTs are largely complementary in the treatment of primary and metastatic prostate cancer.

Currently, as the concept and strategy of cancer theranostics further evolves into clinical management of cancer patients, we believe the future development of PSMA-targeting agents is for personalized precision treatment enabled by quantitative imaging using either radiotheranostic or chemotheranostic agents.

## Data Availability

Not applicable.
